# Incidence and risk factors of spinal epidural hemorrhage after spine surgery: a cross-sectional retrospective analysis of a national database

**DOI:** 10.1186/s12891-020-03337-8

**Published:** 2020-05-25

**Authors:** Ji Hyun Park, Sunny Park, Soo An Choi

**Affiliations:** 1 College of Pharmacy, Korea University, 2511 Sejong-ro, Sejong-si, 30019 South Korea; 2 Research Institute of Pharmaceutical Sciences and Technology, Korea University College of Pharmacy, 2511 Sejong-ro, Sejong-si, 30019 South Korea

**Keywords:** Postoperative spinal epidural hemorrhage, Postoperative spinal epidural hematoma, National claims dataset, Spine surgery, Thoromboprophylaxis, Asian population, Incidence, Risk factors

## Abstract

**Background:**

With increasing number of patients undergoing spine surgery, the spinal epidural hemorrhage (SEH) has become a growing concern. However, current studies on SEH rely on case reports or observations from a single center. Our study attempted to demonstrate the incidence rate and risk factors of SEH using a national dataset.

**Methods:**

A total of 17,549 spine surgery cases from the Health Insurance Review and Assessment Service National Inpatient Sample of 2014 were analyzed. After evaluating the incidence of SEH based on severe cases requiring reoperation, a univariate comparison was performed. Variables found to be significant were included in a multivariable analysis model to determine the risk factors.

**Results:**

The incidence of SEH was found to be 1.15% in Korean population, and there were no severe SEH cases. Our analysis confirmed the previous findings that lumbar surgery, intraoperative blood loss, prolonged surgical time, high blood pressure, use of nonsteroidal anti-inflammatory drugs, and concurrent bleeding factors are the risk factors of SEH. Anterior approach showed a protective effect. The use of anticoagulant demonstrated no statistical significance.

**Conclusion:**

Although severe SEH cases were not detected, the incidence of SEH was similar to that reported in literature. Given that SEH is a rare complication of spine surgery and constitutes an important research area that needs to be studied further, our study makes a meaningful contribution based on a rigorous national level sample for the first time and provides the academic circle and health professionals with a reliable evidence of improved clinical outcomes in such cases.

## Background

Globally, spine surgery rates have drastically increased in the last two decades [1]. There are a number of reasons, such as an increased incidence of obesity, degenerative spinal conditions, and an aging population seeking better quality of life, for this increase [2]. Therefore, it has become necessary to meet the challenging goal of improving the success rate of spine surgeries while minimizing postoperative complications.

Spinal epidural hemorrhage (SEH) manifests a bleeding in the epidural space along the spinal canal [3]. Stimulation that results in hemorrhage around the spinal cord may cause SEH; these include trauma, surgeries, tumors, use of anticoagulants, or invasive neurological procedures. SEH is mostly asymptomatic; however, in some cases, it can result in the accumulation of blood in the epidural space that can lead to spinal cord damage, resulting in neurological deficit [4]. Asymptomatic SEH develops in 33–100% of spinal surgery cases and is known to be spontaneously absorbed postoperatively [5, 6]. Symptomatic SEH causing permanent neurologic deficit rarely develops, and the reported incidence rate is between 0 and 3%. As the incidence of symptomatic SEH has been extremely low, most extant studies on the topic are case studies based on individual clinics [7, 8]. Far less is known on the Asian population including South Korea and Japan regarding all types of SEH, and even the exclusive diagnostic code for SEH has not yet been established [9, 10].

Although there is no consensus with regard to the risk factors, postoperative anticoagulant use is known to increase the incidence of SEH [11, 12]. It is strongly recommended that patients undergoing hip or knee arthroplasty receive anticoagulants to prevent thromboembolic events; Thromboembolic events (TEs) refers to all related events which a blood clot formed in vessel slows or plugs the blood stream [13]. However, this is not the case for patients undergoing spine surgery [14]. The clinical guidelines reveal that there is insufficient evidence to decisively predict the level of risk that prophylaxis could carry; moreover, they identify patients whose benefits outweigh the risks. Consequently, anticoagulant use in patients undergoing spine surgery is left to the discretion of clinicians [15, 16].

More issues are to be resolved in Asian population who had rarely developed thromboembolism after operation [17]. Recent studies have found that the use of anticoagulants in Asian populations is much less than necessary, although the incidence of postoperative TEs in the Asian population is comparable to that in the Western population [18, 19]. With the conventional clinical practice of anticoagulant use combined with the fear of developing severe SEH, clinicians are more likely to avoid anticoagulants [20].

Considering this, a study that attempts to analyze a large dataset created by utilizing postoperative hemorrhage treatment codes on Asian population could clarify the epidemiology of epidural hemorrhage in patients undergone spine surgery. Therefore, the two main goals of this study were: to investigate the incidence of SEH by analyzing a national database, the Health Insurance Review and Assessment Service National Inpatient Sample of 2014 (HIRA-NIS-2014) and to determine the risk factors that could clearly indicate the cause of SEH development in Korean population.

## Methods

### National Inpatient Sample of 2014

South Korea has a National Health Insurance System (NHIS) that covers approximately 98% of the overall Korean population, and 99% of National Health Insurance (NHI) claims data have been electronically reported since 2005. The claims data of Health Insurance Review and Assessment Service (HIRA) pertains to patients’ diagnosis, treatment, surgical history, procedures performed, and prescription drug information. However, the extensive volume and complicated structure of the claims data limit research approaches. To improve accessibility for researchers to use the claims data, HIRA has established the patient sample data sets that have been validated by five different validity tests [21].

These patient sample datasets are as follows: adult patient group, pediatric patient group, all patient group, and inpatient group. The adult patient sample represents approximately 1 million patients aged > 65 years annually (20% of the original data), whereas the pediatric patient sample takes into account 1.1 million patient aged < 20 years annually (10% of the original data). All patient samples represents overall 1.4 million patients annually, which represents 3% of the original population. Lastly, national inpatient sample pertains to 700,000 inpatients (13%), and approximately 400,000 outpatients (1%) annually. All sample dataset contain sampling weights allow obtaining estimated national population. With a 95% concordance, the estimated population and the actual population demonstrated a high level of representativeness [22].

Our study analyzed the customized NHIS dataset of HIRA National Inpatient Sample of 2014 (HIRA-NIS-2014), which contains claims data from January 1, 2014 to December 31, 2014 [23]. This period is defined as the research period in this study. Sampling weights were applied to give best representative estimation of the actual population. The analysis in this study was based on the number of pertinent cases, instead of the number of patients, as one patient might undergo multiple spinal surgeries during the research period. For example, if a patient underwent two different spine surgeries during their independent hospitalizations, the detected number of pertinent cases would be two.

### Availability of data and materials

The data that support the findings of this study are available from HIRA but restrictions apply to the availability of these data, which were used under license for the current study. Thus, these data are not publicly available. However, the data are available from HIRA upon reasonable request and with permission.

### SEH case identification

In Korea, currently there are no disease/treatment codes that exclusively represent SEH. Based on existing studies, this study converted the diagnostic code for postoperative SEH (998.12) indicated in the International Statistical Classification of Diseases and Related Health Problems, 9th revision (ICD-9) to the most appropriate disease code in the 6th Korea Informative Classification of Disease (KOICD-6) codes [24]. As KOICD codes does not differentiate postoperative hematoma from hemorrhage, SEH cases in this study included both spinal epidural hemorrhage and spinal epidural hematoma.

Based on code selection recommendations from the Centers for Disease Control and Prevention/National Center for Health Statistics and KOICD, we first extracted all cases of postoperative hemorrhage and hematoma (T81 and T810), and then narrowed down to severe SEH. Severe SEH cases were defined as the ones that required medical treatment codes for the removal or drainage of hematoma in the central nervous system [9, 25]. T81 and T810 are defined as hemorrhage and hematoma which excludes adverse effect of drug, hematoma of obstetric wound, or failure of transplanted grafts. If the SEH case also had hemorrhage controlling or hematoma drainage codes such as S4755, S4756 or S4754, it would be included in the group of severe SEH cases. Detailed information on the disease and treatment codes were listed in Supplementary Table 1.

Next, all selected cases were individually investigated by three different medical professionals for concurrent diseases, treatment provided, and medications to demonstrate detailed information on severe SEH after spine surgery during patient hospitalizations. Subsequently, all claims records of selected patients during the study period, the disease/treatment codes for the complications of surgical and medical care and postoperative disorders of the nervous system after spine surgery were examined. Detailed information on the disease and treatment codes is listed in Supplementary Table 1.

As an illustration, in a patient with spinal disease undergoing spine surgery, the incidence of postoperative hemorrhage and hematoma was first examined. Then, the intervention for postoperative SEH or spinal nerve compression was evaluated throughout the study period. If any of the codes indicated postoperative disorders of the nervous system or complication from surgical care, every detail of the case was flagged for further review. All variables for a pertinent case were relevant to the hospitalization period. Detailed information on the SEH codes is presented in Supplementary Table 1.

### Literature review for risk factors and spine surgery case selection

To construct the conceptual framework of analysis, the extant studies on incidence and risk factors of SEH were extensively reviewed. The terms “(spinal epidural hematoma) AND (spine surgery) AND (risk factor)” were searched in the MEDLINE database. Simultaneously, the codes for spinal disease were referenced by literature review. In case a patient had a spinal disease code and underwent surgery in relation to the main disease, the patient was considered to have undergone spine surgery. In addition, the risk factors considered in this study were based on the results of the literature review. For example, the definition of extended surgery hours (≥ 2 h) and loss of blood were based on the literature search. The use of nonsteroidal anti-inflammatory drugs (NSAIDs) was defined as the administration of NSAIDs at any time during the hospitalization. The total medical cost was the total expenditure on the hospitalization in a case that was claimed by the medical institution. Insurance types were categorized as the NHI and Medical Aid/Veterans Medical Benefits. Blood loss was calculated by the blood transfusion claims codes of the case. Type of hospitals was classified into two categories: general hospitals (e.g., tertiary academic hospitals and general hospitals) and small hospitals (e.g., hospitals and clinics). The location of hospitals was divided into three categories: metropolitan area (Seoul), other metropolitan areas(e.g., Busan, Daegu, Incheon, Gwangju, Daejon, Ulsan and Sejong), and rural area (all other locations). The duration of hospital stay was calculated by subtracting the admission date from the discharge date.

In case a patient record included any invasive procedures such as lumbar puncture, myelography, and epidural anesthesia, then it was considered that the patient underwent invasive procedures. If a patient had thrombocytopenia or coagulation factor deficiency, the patient was regarded to have a bleeding factor. Trauma case was determined if the patient was admitted through the emergency department.

### Statistical methods and data analyses

After establishing the analytical dataset containing the variables identified as risk factors, we conducted a descriptive analysis. A pairwise correlation test was applied to determine the level of correlation among the set of variables included in the study model. A chi-square test and independent two-sample t-test were used accordingly to determine if there was any significant association between a set of independent variables and both categorical and continuous dependent variables. As the final step, a multivariate logistic regression was utilized prior to which multicollinearity was examined by referring to the variance inflation factor. Data with a variance inflation factor of > 5 were excluded from the model. In the multivariate logistic regression model, this study tries to identify factors that are associated with the incidence of SEH. Sampling weight was used to obtain better estimation of the original population. All statistical analyses were performed using Stata 14.0 [26].

## Results

### Spine surgery and SEH case estimation

**Fig. 1:**
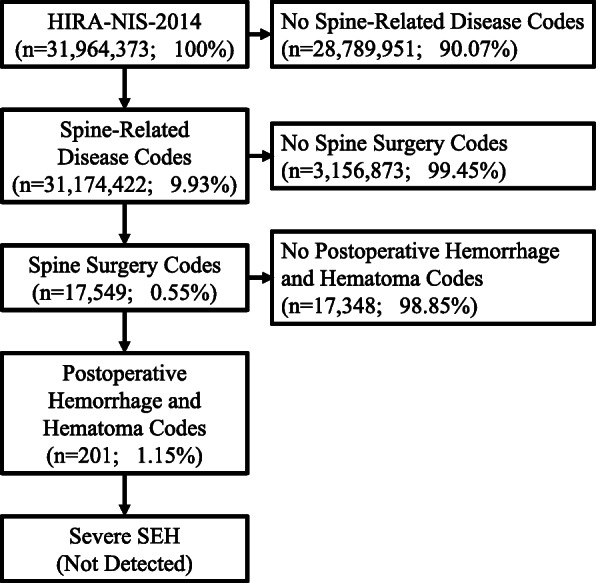
Case selection

Based on the inpatient records, out of a total of 31,964,373 cases, there were 17,549 cases of spinal surgery in 2014. As shown in Fig. 1, the total number of cases of hemorrhage and hematoma occurring after spine surgery was 201 (1.15%), and there were no severe SEH cases as assessed by the codes of hemorrhage evacuation, decompression, or postoperative complication caused by spine surgery.

### SEH risk factors suggested by recent research

Based on the extensive review of recent studies, Table 1 presents the incidence rate and risk factors of SEH. The incidence rate varied from 0 to 23.6% owing to the differences in diagnoses, detection methods, and sample sizes. The identified risk factors are as follows: obesity, hypertension, diabetes, bleeding factors, use of anticoagulant/antiplatelet medications, alcohol consumption, malignancy, previous surgeries, female sex, surgery type (lumbar) and approach, age of 65 and above years, use of NSAIDs, blood loss, trauma, prolonged surgical time, and invasive procedures.

**Table 1:** Incidence rate and risk factors in previous literature

Authors	Year	Incidence (cases/total population)	Risk factors	***P***-value
Kou et al. [27]	2002	NA	Multilevel procedure	0.037
Anno et al. [28]	2019	0.42% (14/3371)	Presence of preoperative coagulopathy	< 0.001
		Cervical: 0.47% (4/856)	Age	NA
		Thoracic: 0.60% (2/332)	Multilevel procedure	NA
		Lumbar: 0.37% (8/2183)	Scar tissue due to previous spinal surgery	NA
			Heparinization	NA
			Vessel damage	NA
Kebaish et al. [29]	2004	0.2%	NA	NA
Yamada et al. [30]	2015	0.39% (32/8250)	≥ 50 mmHg increase in blood pressure after extubation	0.03
			Obesity	0.018
Agnelli et al. [31]	1999	0% (0/15)		
Gerlach et al. [32]	2004	0.7% (13/1954)	Thrombocytopenia	NA
			Anticoagulant use	NA
			Antiplatelet agent use	NA
			Alcohol consumption	NA
			Coagulopathy	NA
			Malignancy	NA
Uribe et al. [33]	2003	0.22% (9/4018)	Thrombocytopenia	NA
			Coagulation factor deficiency	NA
			Medications that predispose to bleeding	NA
			Previous surgery with attendant scarring at the site of epidural hematoma	NA
			Diabetes	
Kim et al. [34]	2019	23.6% (94/304; diagnosed by MRI)	Anticoagulant use	< 0.001
		1.97% (6/304; hematoma evacuation surgery)	Female sex	0.012
			Old age (> 70 years)	0.025
			Use of intraoperative water infusion pump	0.003
			Type of operation	0.01
Ohba et al. [35]	2017	0.468% (6/1282)	Hypertension	NA
			Age > 60 years	NA
			Anticoagulant use	NA
			Use of nonsteroidal anti-inflammatory drugs (NSAIDs)	NA
			Previous spine surgery	NA
			Multilevel procedure	NA
			Blood loss > 1 L	NA
			Infections	NA
Goldstein et al. [36]	2013	1.5% (8/529)	Use of nonsteroidal anti-inflammatory drugs (NSAIDs)	0.24
			Increase in Charlson Comorbidity Index	0.003
Yi et al. [37]	2006	0.28% (9/3270)	Coagulopathy	
			Anticoagulant use	
			Cancer	
Thiele et al. [38]	2008	NA	Predisposed bleeding	NA
			Prolonged surgical time	NA
			Old age	NA
			Trauma	NA
Knusel et al. [39]	2019	0.27% (206/75,878)	Old age	NA
			Obesity (BMI > 35)	NA
			Transfusion	NA
			Multilevel procedure	NA
			Invasive procedure	NA
			Microscope use	NA

*NA* not available

### Characteristics of cases

Patient characteristics were obtained from the 17,549 spinal surgery cases (201 patients with SEH and others). The values for age, total medical cost, and duration of hospital stay were greater for SEH cases than for non-SEH cases. Lumbar spine surgery type and posterior approach showed significance in the univariate analysis. Of all SEH cases, 199 involved treatment with NSAIDs. Among all patients with SEH, 14 underwent surgery for > 2 h in contrast to 187 patients who underwent surgery for ≤2 h. Regarding blood loss, 135 patients were reported to have no blood loss in the claims dataset, and 41 had < 0.5 L of blood loss in the SEH group. Others experienced a blood loss of > 0.5 L (*N* = 25). Most spine surgery procedures wherein SEH occurred (*N* = 139) were performed at small hospitals, whereas only few such procedures (*n* = 63) were performed in general hospitals. The results of the detailed descriptive analyses are shown in Table 2.

**Table 2:** Case demographic and disease characteristics

Characteristics	No SEH	SEH	Total
**Number of Cases**	17,348	201	17,549
**Age** (mean (SD) in year)	56.72 (15.12)	59.08 (13.71)	56.75 (15.11)
**Sex**			
Male	8730	90	8820
Female	8618	111	8729
**Total Medical Payment**^**a**^ (mean (SD) in USD)	2438 (2071)	3028 (2099)	2445 (2072)
**Duration of Hospital Stay** (mean (SD) in days)	13.41 (9.49)	19.35 (11.66)	13.48 (9.54)
**Insurance Types**			
National Health Insurance	16,278	185	16,463
Medical Aid & Veterans	1070	16	1086
**Spine Surgery Types** (%)			
Lumbar	12,381 (98.64)	170(1.35)	12,551 (100)
Not Lumbar	4967 (99.38)	31 (0.62)	4998 (100)
**Spine Surgery Approach** (%)			
Anterior	3918 (98.47)	61 (1.53)	3979 (100)
Not Anterior	13,430 (98.97)	140 (1.03)	13,570 (100)
**Infections**			
No	15,983	180	16,163
Yes	1365	21	1386
**Diabetes**			
No	15,629	177	15,806
Yes	1719	24	1743
**Hypertension**			
No	11,972	121	12,093
Yes	5376	80	5456
**Use of NSAIDs**			
No	803	2	805
Yes	16,545	199	16,744
**Invasive Procedures**^b^ (%)			
No	13,056 (98.89)	146 (1.11)	13,202 (100)
Yes	4292 (98.73)	55 (1.27)	4347 (100)
**Bleeding Factors**^c^			
No	14,223	140	14,363
Yes	3125	61	3186
**Trauma**			
No	16,499	188	16,687
Yes	849	13	862
**Anticoagulant Use**			
No	15,090	178	15,268
Yes	2258	23	2281
**Surgery Hours**			
0 < HR ≤ 2	17,149	187	17,336
Over 2 h	199	14	213
**Blood Loss**			
No Loss	14,526	135	14,661
0 < Loss ≤0.5 L	1871	41	1912
Over 0.5 L	951	25	976
**Type of Hospitals**^**d**^ (%)			
General Hospitals	6371 (99.04)	62 (0.96)	6433 (100)
Small Hospitals	10,977 (98.93)	139 (1.07)	11,116 (100)
**Number of Beds in Hospitals** (%)			
Beds ≤200	10,611 (98.99)	108 (1.01)	10,719 (100)
More than 200 Beds	6737 (98.64)	93 (1.36)	6830 (100)
**Location of Hospitals** (%)			
Metropolitan area (Seoul)	5066 (99.37)	32 (0.63)	5098 (100)
Other Metropolitan areas	8853 (99.05)	86 (0.95)	8939 (100)
Rural Area	3421 (97.63)	83 (2.37)	3504 (100)
**Total (cases)**			

*SEH* spinal epidural hemorrhage and hematoma, *NSAIDs* non-steroidal anti-inflammatory drugs

^a^Total medical payments: The sum of the payments that patients pay for all medical services when they leave from a hospital

^b^Invasive procedures: lumbar puncture, myelography, and epidural anesthesia

^c^Bleeding factors: coagulation factor deficiency and thrombocytopenia

^d^Type of Hospitals: The hospitals are classified into four categories in Korea, based on their function and size. From largest to smallest, they are tertiary general hospital, general hospital, hospital, and clinic. Small hospitals include hospital and clinic. General hospitals includes tertiary general and general hospitals

### SEH risk factors

A pairwise correlation analysis that was performed before the univariate analysis revealed that there was no significant correlation among the independent variables. In the univariate analysis, we found that most independent variables that have been included in the study model were associated with the incidence rate of SEH. The following variables were found to have a statistically significant relationship with SEH. Age (*p* = 0.03), total medical cost (*p* < 0.001), lumbar spine surgery (*p* < 0.001), anterior-approach surgery (*p* < 0.001), hypertension (*p* = 0.01), use of NSAIDs (*p* = 0.01), bleeding factors (*p* < 0.001), surgical time >  2 h (*p* < 0.001), blood loss > 0.5 L (*p* < 0.001), number of hospital beds > 200 (*p* = 0.03), and hospitals located in the rural areas. The variables with marginal significance (0.05 ≤ *p* < 0.20), such as sex, infections, and small hospital types, were also included in the multivariable logistic regression model. The use of anticoagulant was not found to be a significant risk factor as per the univariate analysis, however, we included the variable in the multivariate analysis since other extant studies demonstrated it as a strong risk factor of SEH. Table 3 presents the results of the univariate analysis. Lumbar spine surgery, blood loss > 0.5 L, surgery > 2 h, hypertension, use of NSAIDs, presence of bleeding factors, small hospitals, and location of the hospital were identified as risk factors for SEH. However, anterior approach and anticoagulant use showed a protective effect on SEH incidence. The analyses of the survey data included the calculation of sampling weights, which ensured about 95% of concordance of the patient sample dataset with the actual population [21]. The details on the results of the multivariate analysis are presented in Table 4. The analysis results from the non-weighted dataset are provided in Supplement Table 2.

**Table 3:** Univariate analysis result of variables

Variables	***P***-value*
Age	0.03
Sex	0.12‡
Total medical cost	0.00
Duration of hospital stay	0.00
Insurance types	0.24†
Spine surgery type: lumbar	0.00
Spine surgery approach: anterior	0.00
Infection	0.18‡
Diabetes	0.34†
Hypertension	0.01
Use of NSAIDs	0.01
Invasive procedures	0.39†
Bleeding factors	0.00
Trauma	0.31†
Anticoagulant use	0.51†
Surgical time: > 2 h	0.00
Blood loss: > 0.5 L	0.00
Type of hospital: small hospitals	0.09‡
Number of hospital beds: > 200	0.03
Location of hospital: rural area	0.00

*Variables with a dagger (†) mean that the *P*-value of the corresponding variable is not statistically significant. Variables with a double dagger (‡) means that the *P*-value of the corresponding variable is located between 0.05 and 0.20, indicating marginal significance. A *P*-value of 0.00 indicates a *P*-value < 0.001

**Table 4:** Multivariable logistic regression results

Risk factors	***P*** > |z|*	OR (95% CI)
Spine surgery approach: anterior	0.03	0.43 (0.20–0.90)
Spine surgery type: lumbar	0.01	1.75 (1.15–2.65)
Blood loss: > 0.5 L	0.00	2.11 (1.31–3.41)
Surgical time: > 2 h	0.00	7.22 (3.82–13.67)
Hypertension	0.03	1.41 (1.04–1.90)
Use of NSAIDs	0.01	7.30 (1.70–31.44)
Bleeding factors	0.00	1.92 (1.41–2.62)
Anticoagulant use	0.08	0.66 (0.42–1.05)
Type of hospital: small hospitals^a^	0.03	1.48 (1.05–2.08)
Location of hospital: rural area	0.00	3.11 (2.32–4.18)

*OR* odds ratio, *CI* confidence interval

*P-value of 0.00 is *p*-value < 0.001.

^a^In the Korean health care system, the hospitals are classified into four categories, based on their function and size. From largest to smallest, they are tertiary general hospital, general hospital, hospital, and clinic. Small hospitals include hospital and clinic

## Discussion

### SEH case estimation

This is the first study to utilize a national database of Korean population to investigate the incidence and risk factors of SEH. Of total 17,549 cases of spine surgery, 201 (1.15%) were found to be the case of symptomatic SEH, and there was no case of severe SEH detected leading to continuous nerve damage and reoperation.

So far, only few studies have assessed the topic of SEH in the Asian population [12, 40–43], where the reported incidence of SEH with nerve deficit ranges from 0.1 to 0.9%. Imajo et al. utilized a series of nationwide survey results on the complications among Japanese patients who underwent spine surgery from 1994 to 2001 (*N* = 31,380) and reported SEH incidence rate of 0.9%, whereas Anno et al. reported an incidence rate of 0.4% based on data from a single-center [43]. They found that SEH had a positive association with prolonged surgical time, intraoperative blood loss, and old age. The lumbar procedure was also identified as a risk factor in a previous study, and the neurological outcome was found to be poor in two cases.

Yamada et al. reported that postoperative SEH evacuation was performed in 32 of 8250 patients at their institution, out of which 14 patients developed severe paralysis [44]. The patients who underwent SEH evacuation within 24 h of the onset of symptoms had better clinical outcomes. The duration of onset for SEH symptoms ranged from 3 h to 3 days, and the median duration was 24 h after the initial surgery. The duration of SEH evacuation ranged from 2 h to 5 days after the onset of the symptoms. Yi et al. analyzed 3720 spine surgery cases over a period of 7 years (1998–2005) at a single institution in Korea and reported nine cases (0.24%) of severe neurologic deterioration requiring surgical decompression [42]. Lumbar spine surgery, anticoagulation therapy, and highly vascularized tumor were also reported as the risk factors of SEH. Better clinical outcomes have been consistently noted in patients who undergo early intervention to evacuate SEH.

There were no cases of reoperation for hemorrhage removal or neurological deterioration due to SEH in our analytic dataset, which could be explained based on the following reasoning. In Korea, the period of hospitalization after spine surgery is more than three-fold longer than that in other countries [45]. The average duration of hospital stay for our patient sample was 13.48 days, which is more than three-fold longer than the international average of 4 days [46]. The SEH group showed a longer hospital stay at 19.35 days, and the medical care team might have had adequate time to detect the potential development of epidural hemorrhage. Therefore, postoperative hemorrhage and hemorrhage were estimated to develop in 1.15% of the total patients; this incidence is higher than that in similar ethnic groups based on the literature. However, the incidence of severe SEH requiring reoperation was minimal.

Our analysis of the risk factors of SEH confirmed previous findings that the anterior-approach, lumbar surgery, intraoperative blood loss, prolonged surgical time, high blood pressure, use of NSAIDs, and concurrent bleeding factors are associated with SEH. Moreover, the type and location of the hospital were also found to be associated with the risk of SEH, which could suggest that the differences in the medical service quality based on the location of the hospitals could lead to different surgical outcomes. Inconsistent quality of inter-institution and inter-nation medical care has been a major global concern [46].

In our univariate analysis, anticoagulant use was an insignificant risk factor of SEH (*p* = 0.51). However, the use of anticoagulant and/or antiplatelet agents has been considered as one of the most important triggers for SEH incidence as reported previously [8, 42, 47, 48]. Subsequently, the use of anticoagulant was incorporated in the multivariate analysis to demonstrate its effects on postoperative hemorrhage and hematoma, which showed no statistical (*p* = 0.08; OR = 0.66 [0.42–1.05]).

Postoperative thromboprophylaxis is applied only to 7.89% of patients undergoing spine surgery in Korea compared to 56.3% of the patients in North America. The apprehension against the use of anticoagulant because of suspected incidence of severe SEH might be the reason for the differences in the results in our analysis. However, thromboprophylaxis with optimal dose has been known to have no negative effect on the incidence of SEH [49]. As the preparation of thromboprophylaxis protocol has been incorporated into the hospital accreditation evaluation in Korea, standardized and optimized thromboprophylaxis might be considered in qualified patients without contraindication [19].

Our study attempts to fill the existing gap in the data on SEH. This is the first study examining the risk factors of SEH based on a national insurance database. Previous analyses and reports were based on single-center data and on an availability basis. Consequently, the findings of our study on the risk factors of SEH could help clinicians make suitable clinical decisions for patients undergoing spine surgery. Moreover, these data from this pilot study can be utilized for further studies on SEH and for the development of an exclusive disease code for SEH.

Although our study makes a significant contribution to the literature, the absence of an exclusive disease code for SEH in KOICD-6 is its limitation. The study attempted to overcome this constraint that exists in the national insurance database by utilizing disease/treatment codes and interventions for severe cases of SEH. Although the HIRA-NIS dataset is derived from the national claims data, it only contains information on treatments being used during hospitalization, and does not provide the exact administration time. Further studies focused at bridging these gaps are strongly encouraged.

## Conclusions

We analyzed the incidence and the risk factors of SEH after spine surgery in patients in Korea by utilizing rigorous nationwide insurance data for the first time. Given that SEH is a rare complication of spine surgery and an important research area that is yet to be studied, our study makes a meaningful contribution to both the academic and medical fields by identifying the risk factors of SEH. Based on the major findings of our study, the use of anticoagulant showed no significant effect on the incidence of SEH. However, extended surgery time, intraoperative blood loss, use of NSAIDs, and preexisting bleeding factors need to be carefully evaluated in patients undergoing spinal surgery. Additionally, further studies to obtain more knowledge regarding any additional risk factors and incidence rate of SEH are warranted.

## Supplementary information

**Additional file 1: Table S1.** Disease or treatment codes. **Table S2.** Multivariable logistic regression results (Non-Weighted).

## Data Availability

The data that support the findings of this study are available in HIRA, but restrictions apply to the availability of these data, which were used under license for the current study. Thus, these data are not publicly available. However, the data are available from HIRA upon reasonable request and with permission.
