# Supplementary material for: Incidence and risk factors of spinal epidural hemorrhage after spine surgery: a cross-sectional retrospective analysis of a national database
Source: BMC Musculoskelet Disord. 2020 May 25;21:324. doi: 10.1186/s12891-020-03337-8 (PMC7249427; doi:10.1186/s12891-020-03337-8)
Supplement: Supplementary file 1 — Additional file 1: Table S1. Disease or treatment codes. Table S2. Multivariable logistic regression results (Non-Weighted). [file 12891_2020_3337_MOESM1_ESM.docx]

| **Table S1. Disease or treatment codes** | |
| --- | --- |
|  | **Disease or treatment codes** |
| Spinal disease codes | M40-M54 |
|  | S12, S120–S122, S127, S129 |
|  | S13, S130–S132, S133, S134 |
|  | S14, S140–S142 |
|  | S22, S220–S242 |
|  | S32, S320–342, S343, S344 |
|  | Q005, Q006, Q675, Q76, Q760–Q764, Q777–Q779 |
|  | M99X1–M99X3, M995, M996, M997 |
| Postoperative hematoma and hemorrhage | T81, T810 |
| Evacuation of hematoma or decompression | S4755, S4756, S4594 |
| Postprocedural disorders of the nervous system | G97, G978, G979 |
| Complication of surgical and medical care | T888, T889, T983 |
|  | Y83, Y834, Y836, Y838, Y839, Y88, Y883 |
|  |  |

| **Table S2. Multivariable logistic regression results (Non-Weighted)** | | |
| --- | --- | --- |
| **Risk factors** | **P > \|z\|*** | **OR (95% CI)** |
| Spine surgery approach: anterior | 0.01 | 0.37 (0.17–0.79) |
| Spine surgery type: lumbar | 0.04 | 1.54 (1.01–2.37) |
| Blood loss: > 0.5 L | 0.00 | 1.89 (1.36–2.62) |
| Surgical time: > 2 h | 0.00 | 7.44 (4.11–13.47) |
| Hypertension | 0.04 | 1.37 (1.01–1.86) |
| Use of NSAIDs | 0.07 | 3.81 (0.90–16.11) |
| Bleeding factors | 0.00 | 1.87 (1.37–2.54) |
| Anticoagulant use | 0.08 | 0.66 (0.40–1.05) |
| Type of hospital: small hospitals† | 0.03 | 1.51 (1.08–2.13) |
| Location of hospital: rural area | 0.00 | 3.14 (2.35–4.19) |
| OR, odds ratio; CI, confidence interval | | |
| *P-value of 0.00 is p-value < 0.001. | | |
| †In the Korean health care system, the hospitals are classified into four categories, based on their function and size. From largest to smallest, they are tertiary general hospital, general hospital, hospital, and clinic. Small hospitals include hospital and clinic. | | |

**References**

1. Quan H, Sundararajan V, Halfon P, et al. Coding algorithms for defining comorbidities in ICD-9-CM and ICD-10 administrative data. Medical care. 2005:1130-1139.

2. Awad JN, Kebaish KM, Donigan J, Cohen DB, Kostuik JP. Analysis of the risk factors for the development of post-operative spinal epidural haematoma. The Journal of bone and joint surgery British volume. 2005;87(9):1248-1252 DOI: 10.1302/0301-620X.87B9.16518.

3. Cox JB, Weaver KJ, Neal DW, Jacob RP, Hoh DJ. Decreased incidence of venous thromboembolism after spine surgery with early multimodal prophylaxis: Clinical article. J Neurosurg Spine. 2014;21(4):677-684 DOI: 10.3171/2014.6.SPINE13447.

4. Korean Information Center for Disease. Disease codes status of admission and outpatient treatment Aug 16, 2018. http://www.koicd.kr/2016/stats/diseaseStats.do. Accessed Aug 16, 2018.

5. Rogers SO, Kilaru RK, Hosokawa P, Henderson WG, Zinner MJ, Khuri SF. Multivariable Predictors of Postoperative Venous Thromboembolic Events after General and Vascular Surgery: Results from the Patient Safety in Surgery Study. Journal of the American College of Surgeons. 2007;204(6):1211-1221 DOI: https://doi.org/10.1016/j.jamcollsurg.2007.02.072.
